# Titers of IgG and IgA against SARS-CoV-2 proteins and their association with symptoms in mild COVID-19 infection

**DOI:** 10.1038/s41598-024-59634-y

**Published:** 2024-06-03

**Authors:** Andrés G. Abril, Jose Alejandre, Anais Mariscal, Leticia Alserawan, Nuria Rabella, Eva Roman, Joaquin Lopez-Contreras, Ferran Navarro, Elena Serrano, Josep F. Nomdedeu, Silvia Vidal

**Affiliations:** 1 Departament Biologia Cel·lular, Facultat de Medicina, Fisiologia i Immunologia, Universitat Autònoma de Barcelona, Cerdanyola del Vallès, 08193 Bellaterra, Spain; 2 Althaia Xarxa Assistencial Universitària de Manresa, 08243 Manresa, Spain; 3 Institut de Recerca i Innovació en Ciències de la Vida i de la Salut a la Catalunya Central (IRIS-CC), 08500 Vic, Spain; 4 Grup de Malalties Inflamatòries, IIB-Sant Pau, Institut Recerca Hospital de la Santa Creu i Sant Pau, 08041 Barcelona, Spain; 5 Servei d’Immunologia, Hospital de la Santa Creu i Sant Pau, 08041 Barcelona, Spain; 6 Servei de Microbiologia, Hospital de la Santa Creu i Sant Pau, 08041 Barcelona, Spain; 7 Servei de Patologia Digestiva, Hospital de la Santa Creu i Sant Pau, 08041 Barcelona, Spain; 8 Servei de Malalties Infeccioses, Hospital de la Santa Creu i Sant Pau, 08041 Barcelona, Spain; 9 Biobanc IIB-Sant Pau, 08041 Barcelona, Spain; 10 Servei d’Hematologia, Hospital de la Santa Creu i Sant Pau, 08041 Barcelona, Spain

**Keywords:** COVID-19, SARS-CoV-2, Seroprevalence, IgG, IgA, Infection, Adaptive immunity

## Abstract

Humoral immunity in COVID-19 includes antibodies (Abs) targeting spike (S) and nucleocapsid (N) SARS-CoV-2 proteins. Antibody levels are known to correlate with disease severity, but titers are poorly reported in mild or asymptomatic cases. Here, we analyzed the titers of IgA and IgG against SARS-CoV-2 proteins in samples from 200 unvaccinated Hospital Workers (HWs) with mild COVID-19 at two time points after infection. We analyzed the relationship between Ab titers and patient characteristics, clinical features, and evolution over time. Significant differences in IgG and IgA titers against N, S1 and S2 proteins were found when samples were segregated according to time T1 after infection, seroprevalence at T1, sex and age of HWs and symptoms at infection. We found that IgM + samples had higher titers of IgG against N antigen and IgA against S1 and S2 antigens than IgM − samples. There were significant correlations between anti-S1 and S2 Abs. Interestingly, IgM + patients with dyspnea had lower titers of IgG and IgA against N, S1 and S2 than those without dyspnea. Comparing T1 and T2, we found that IgA against N, S1 and S2 but only IgG against certain Ag decreased significantly. In conclusion, an association was established between Ab titers and the development of infection symptoms.

## Introduction

Severe Acute Respiratory Syndrome Coronavirus 2 SARS-CoV-2 infection was first reported in China in 2019, and World Health Organization declared COVID-19 a pandemic on 11 March 2020. Scientific efforts to understand and control this new infection have focused not only on the discovery of optimal treatments and vaccines to reduce the clinical impact and spread of the disease, but also on gaining an understanding of the interplay between the new virus and the immune system^1,2^. In the first wave of the pandemic, incubation periods lasted from three to seven days^3^. Fever, cough, and fatigue were the most common symptoms, whereas nasal congestion, runny nose, and diarrhea were only reported in a small proportion of patients. Severe cases progressed rapidly to acute respiratory distress syndrome, shock, difficult-to-treat metabolic acidosis, bleeding and coagulation dysfunction^3^. The clinical spectrum was wide, leading to a difficult differential diagnosis. Therefore, polymerase chain reaction (PCR)–based viral RNA detection has become the main tool to confirm a diagnosis of SARS-CoV-2 infection in practice worldwide^4^. Appropriately designed seroprevalence studies have provided estimates of the infected proportion of a population^5^. This kind of study had the advantage of accounting for asymptomatic cases^6^. Infection with SARS-CoV-2 induces humoral and cellular immune responses^7,8^. Humoral immunity includes Abs of several immunoglobulin isotypes targeting SARS-CoV-2 proteins, most notably spike (S) and nucleocapsid (N) proteins^5,6^. The concentration of Abs in blood varies substantially between individuals depending on age, patient characteristics and time since infection^5–9^. Thus, large variations in Ab levels between individuals prevent this variable from being a consistent measure of immunity^5,10^. In addition, the longitudinal follow-up of individuals infected with SARS-CoV-2 suggests a pattern of waning Ab responses consistent with the results obtained with other coronaviruses^9,11^. Reports have shown that an Ab´s response peak is elevated between the second and third week after infection^12^. This peak is characterized by the presence of IgA, IgM and IgG in serum and mucosal fluids^12–15^. Although IgM is the first line of humoral response, one particularity of the SARS-CoV-2 infection is that all three isotypes can be detected in a short period of time^14^. IgG and IgA can frequently be detected even before IgM^12,14^, suggesting that the initial IgM response may be weak. Moreover, specific IgG or IgA B-cell precursors exist in the memory B-cell compartment and class-switching occurs rapidly after antigen encounter. Therefore, the detection of IgG or IgA may be more sensitive than IgM detection in the early stages of infection^14^. It's important to note that the specific role and dynamics of each antibody isotype in a viral infection can vary depending on the type of virus, the site of infection, the duration since infection, and the individual's immune response. IgM, as an early immune response antibody, plays several roles including neutralizing viruses, activating the complement system for an enhanced immune response, and clearing immune complexes. IgG provides long-term immunity, neutralizes viruses, opsonizes them for immune cell recognition, and triggers antibody-dependent cellular cytotoxicity. IgA acts as the first line of defense, providing protection at sites such as the respiratory and gastrointestinal tracts by trapping and immobilizing viruses, regulating inflammation, and promoting a balanced immune response. Although Abs correlate positively with disease severity, titers in mild or asymptomatic cases are poorly reported^10,12,14,15^. Serology is indeed influenced by factors beyond just the time since diagnosis, including viral load and individual characteristics. While we were unable to determine all these factors in the analysis, our primary objective was to investigate the relationship between serological responses and patient characteristics, as well as COVID-19 symptoms. We have paid special attention to seroconversion dynamics in relation to the stage of infection, particularly in the context of COVID-19, where pinpointing the exact moment of infection was challenging. Detection of IgM antibodies typically indicates a relatively recent infection, as IgM levels tend to decline weeks following the initial infection. By stratifying our samples based on IgM positivity, we aimed to explore the relationship between antibody kinetics and the timing of infection onset. In fact, IgM and IgG responses have been used to distinguish acute and convalescent phases. According to a recent study, the seroprevalence of SARS-CoV-2 IgM and IgG in COVID-19 patients was over 70% less than 7 days after symptom onset^16,17^. In this study, we specifically examined the dynamics of IgA and IgG antibody titers against various SARS-CoV-2 proteins in 200 unvaccinated Hospital Workers (HWs) with confirmed mild cases of COVID-19. Our analysis also involved describing the correlation between antibody titers and patient characteristics, clinical features, and the evolution of antibody levels over time. By focusing on the presence of detectable IgM, we aimed to understand the broader patterns of serological response without delving into specific titers. This approach allowed us to explore the relationship between antibody levels and various aspects of the disease in our cohort of HWs.

## Results

### Titers of Abs against SARS-CoV-2 antigens after infection

**Figure 1:**
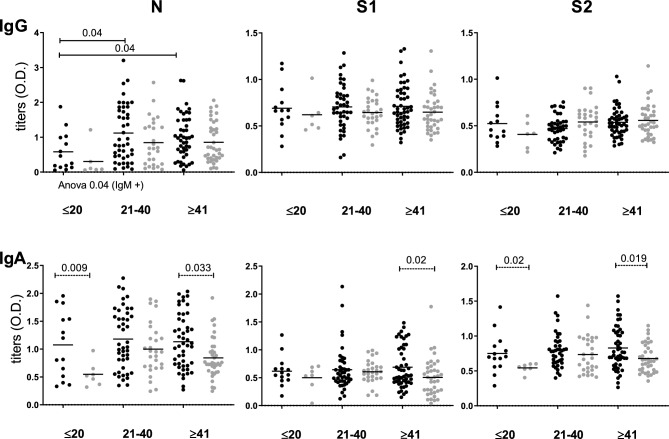
Distribution of IgG and IgA titers according to time since infection (days). Titers were expressed in O.D.. Serum samples from IgM + HWs (black dots) and IgM--—HWs (gray dots) were grouped by time since infection (days) and compared by ANOVA test (significant values are shown under the X axis), followed by correction by the Bonferroni test (significant values shown as bars at the top of each figure). The Mann Whitney test was used to find differences between IgM + HWs titers versus IgM—HWs titers (discontinued lines at the top of each figure). Significant *p* value < 0.05.

Since T1 runs from five to 75 days, we performed a comparative analysis between samples from HWs with and without IgM anti-SARS-CoV2 antibodies against Nucleocapsid (indicated as IgM + vs. IgM −, respectively) without finding any significant difference (Table 1). Fifty-four percent of patients were IgM + at T1 (titer not shown). For the analysis, T1 samples from HWs were arbitrarily grouped according to time since infection in: (a) less than 21d, (b) between 21 and 40d, and (c) more than 40d. A significant proportion of HWs (N = 50 HWs; 51.02%) was still IgM + more than 40d after reported infection (Fig. 1 and Table 1).

**Table 1:** Patients’ characteristics.

	Health Workers (HWs)	N	(%)*	IgM + N (%)	IgM − N (%)	*p*
		200	(100)	108 (54)	92 (46)	
Gender	Male	49	(22.7)	18 (36.73)	31 (63.26)	n.s
	Female	151	(77.3)	90 (59.60)	61 (40.39)	
Time since infection	< 21 days	22	(11)	14 (63.63)	8 (36.36)	n.s
	21–40 days	80	(40)	44 (55.00)	36 (45.00)	
	> 40 days	98	(49)	50 (51.02)	48 (48.97)	
Antecedents	Smoke	50	(25.5)	25 (50)	25 (50)	n.s
	Allergy	23	(12.5)	15 (65.21)	8 (34.78)	
	Hypertension	12	(5.5)	5 (41.66)	7 (58.33)	
	Asthma	13	(6.5)	10 (76.92)	3 (23.07)	
	Diabetes	3	(1.5)	2 (66.66)	1 (33.33)	
	Miscellaneous	37	(17.5)	17 (45.94)	20 (54.05)	
	Not reported	67	(39)	12 (23.07)	45 (76.92)	
Age	(years ± s.e.m.)	43.03 ± 0.83		44.05 ± 1.06	44.34 ± 1.15	n.s

*Proportion of total HWs in each group.

The distribution of patients for each characteristic was compared according to IgM seroprevalence by chi-square and no significant difference was found (n.s.).

When comparing the serology of IgM + and IgM − samples, we found that the titers of IgA against N and S2 in groups of < 21d (*p* = 0.009 and *p* = 0.02 respectively) and IgA against N, S1 and S2 ≥ 41d since infection (*p* = 0.033, *p* = 0.02 and *p* = 0.019 respectively) were higher in IgM + HWs. Then, when samples were grouped as IgM + and IgM − and titers were compared based on the time since infection, only in IgM+ samples were the titers of IgG against N found to differ significantly depending on the time since infection (*p* = 0.04). Without segregating by IgM serology, the titers of IgG against N were significantly lower in < 21d group than the other two groups: 21-40d and ≥ 41d since infection (Supplementary Table 1).

**Figure 2:**
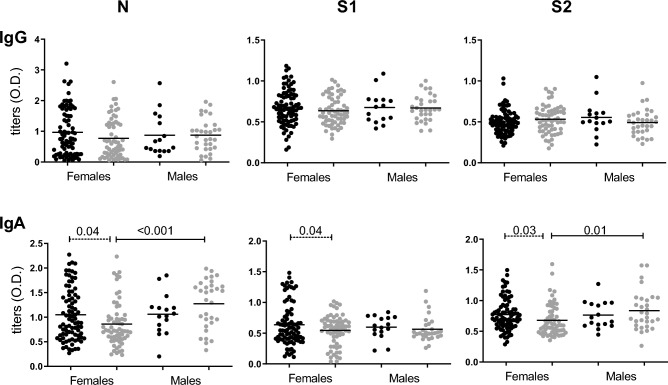
Distribution of IgG and IgA titers according to sex. SARS-CoV-2 Ab titers are expressed as O.D. from serum samples separated by IgM + HWs (black dots) and IgM − HWs (gray dots) and by sex (male vs female). IgM + or IgM—female versus male Ab titers were compared (continuous lines over series with significant *p* values) by Mann Whitney test; also, IgM + vs IgM − males and females were also compared separately (discontinued lines over series). Significant *p* value < 0.05.

Samples from HWs were grouped according to sex (Fig. 2). When comparing IgM + and IgM −, we found that, in females, the titers of IgA against N, S1 and S2 were higher in IgM + samples (*p* = 0.04, 0.04 and 0.03, respectively). Samples were then grouped as IgM + and IgM − and titers were compared according to sex. We found that, in IgM − samples, females had lower titers of IgA against N and S2 (*p* < 0.001 and *p* = 0.01 respectively). Similarly, without segregating by IgM serology, the titers of IgA against N in females were lower than in males (Supplementary Table 1).

**Figure 3:**
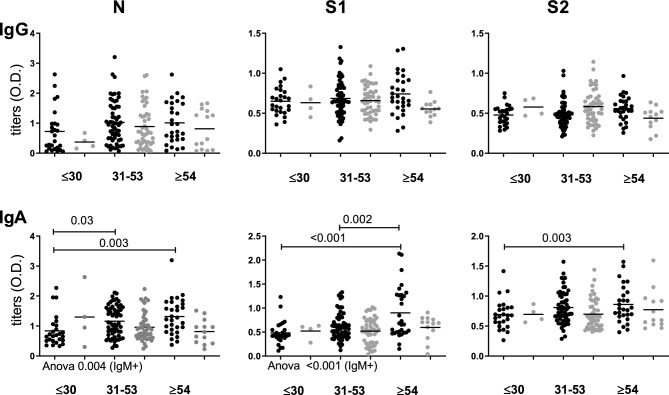
Distribution of IgG and IgA titers according to age. Titers expressed as O.D. from IgM + HWs (black dots) and IgM—HWs (gray dots) were split into three age groups (years old) and compared by ANOVA (significant values are shown under x axis) followed by post hoc Bonferroni test (significant *p* values are shown with continuous bars over dots series). Significant *p* value < 0.05.

Finally, samples from HWs were also divided in three groups of age (a) less than 31 years old; (b) between 31 and 53 years old; and (c) more than 53 years old. We found higher titers in the IgM + HWs of ≥ 54 years old when compared with the group of < 31 years old for IgA against N, S1 and S2 and (*p* = 0.003; *p* < 0.001; *p* = 0.003, respectively). Also, higher titers of IgA against N in the 31–53 years old group compared with < 31 years old IgM + HWs (*p* = 0.03) and IgA against S1 in the ≥ 54 years old group compared with 31–53 (*p* = 0.002). When samples were analyzed separately as IgM + or IgM −, the titers of IgA against N and S1 among IgM + HWs were different depending on the age (*p* = 0.004; *p* < 0.001, respectively) (Fig. 3). Without separating by IgM serology, the titers of IgA against S1 in the oldest group were higher than in the other groups of age (Supplementary Table 1).

No influence of smoking habit on Ab titers was found in this cohort of HWs. However, lower titers of IgG against S2 were observed in HWs who had previously been vaccinated against the flu (N = 68) when compared with non-vaccinated HWs (*p* = 0.01).

The most significant correlations were found between the titers of a) IgG with IgA anti-N; b) IgG anti-S1 and anti-S2; c) IgA anti-S1 and anti-S2 (Table 2). Similar results were found when we correlated IgM + and IgM − samples separately. (Supplementary Table 2).

**Table 2:** Correlation matrix of Ab titers.

	IgG			IgA		
	Antigens	S1	S2	N	S1	S2
IgG	N	0.11	0.076	0.453***	0.147	0.175*
	S1		0.472***	0.085	0.224*	0.289*
	S2			0.085	0.238*	0.296*
IgA	N				0.222	0.288*
	S1					0.471***

Pearson's correlation coefficients are shown, and significant values are highlighted in bold. (***p < 0.001, *p < 0.05).

### Association of infection symptoms with anti-SARS-CoV-2 Abs

**Figure 4:**
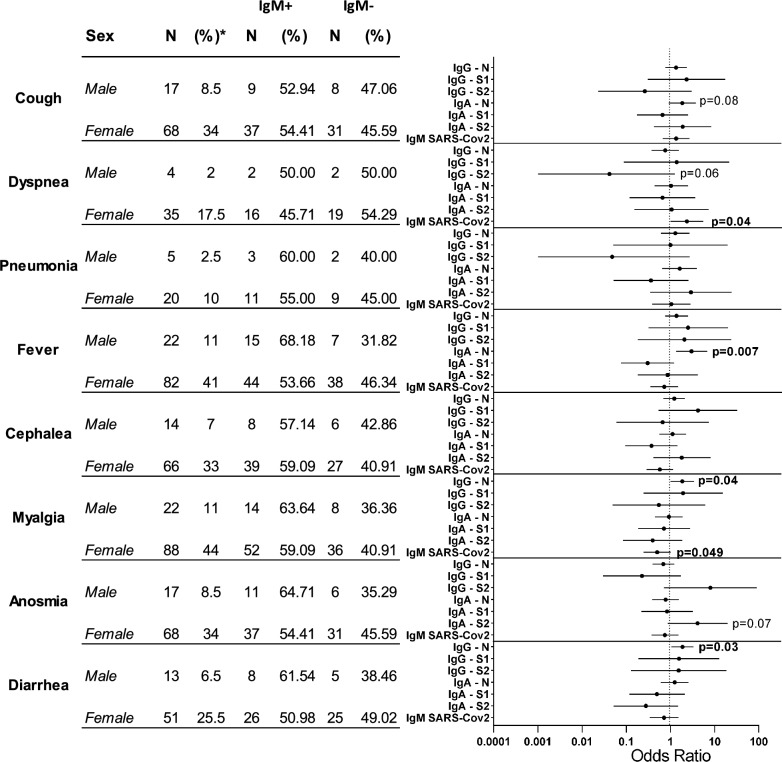
Association of symtoms with serology in COVID19. Frequency of symptoms in patients by sex and the presence of IgM anti-SARS-CoV2 and Forest plot of output from the logistic regression model evaluating odds for each symptom. The variables identified in the final model, along with the corresponding odds ratios and confidence intervals, are shown in the figure.

Infected HWs showed a miscellaneous spectrum of symptoms including fever, cough, dyspnea, anosmia, cephalea, arthralgia, myalgia, fatigue, diarrhea, and skin rash (excluded from analysis because of low frequency) (Fig. 4). The most frequent symptoms were myalgia and fever, followed by cough, anosmia and cephalea. Fifteen HWs showed a combination of these symptoms (female 14; male 1). The least frequent symptoms were diarrhea and dyspnea. Finally, 25 HWs had pneumonia confirmed by radiologic tests. Only five HWs (four females, one male) showed all the symptoms. These five HWs were not smokers, and only one had allergic asthma as a relevant clinical antecedent. No significant differences between male and female HWs were found.

We then compared the titers of different Abs among HWs with and without symptoms after stratifying them based on IgM positivity or negativity (Supplementary Table 3). We found that IgM + and IgM − HWs who suffered fever had higher titers of IgG and IgA anti-N, whereas only IgM − HWs with fever had more IgG anti-S2. We also found that HWs who reported dry cough had higher titers of IgA anti-N, while IgM + HWs with anosmia had less IgG anti-N and S1, and IgA anti-N. Although only a few HWs developed pneumonia in this cohort, we found that IgM + HWs with this symptom had more IgA anti-N. Finally, IgM + HWs with severe diarrhea had higher titers of IgG anti-N. Our results also showed that only IgM + HWs with dyspnea had lower titers of IgG and IgA against N, S1 and S2. Similarly, anosmia in IgM + HWs was associated with lower titers of IgG anti-N and S1 and IgA anti-N. Interestingly, among IgM − HWs, those with anosmia had more IgG anti-S2 and those with pneumonia had less IgG anti-S2. The association of each symptom to antibodies (based on isotype, titer and specificity) was also modeled using logistic regression, treating IgM seroprevalence as just one among the other variables. Figure 4 shows the forest plots of the logistic regression outputs. In the models, IgA anti-N antibodies were associated with fever and cough, IgG anti-N antibodies were associated with diarrhea and myalgia and IgM anti-SARS-CoV2 antibodies were associated with dyspnea.

### Kinetics of specific Abs against SARS–CoV-2 antigens

**Figure 5:**
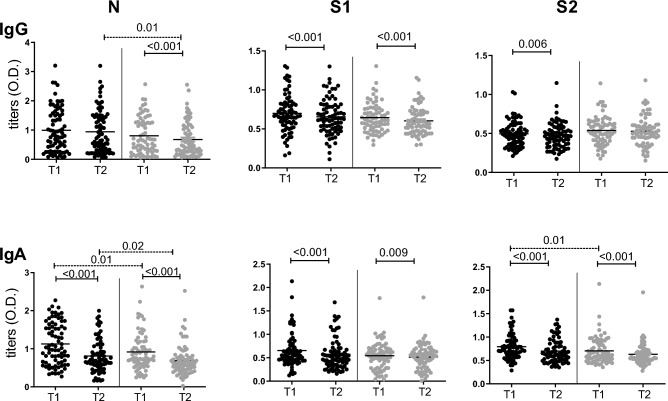
Kinetics of IgG and IgA titers. Titers are expressed O.D. Serum samples from IgM + HWs (black dots) and IgM—HWs (gray dots) were analyzed separately over time. T1 versus T2 are compared by paired T student test (statistically significant *p* values are indicated over continuous capped lines); also, IgM + HWs versus IgM–HWs Ab titers were compared using Wilcoxon test (discontinuous lines). Significant *p* value < 0.05.

We then analyzed the kinetics of IgG and IgA anti-N, S1 and S2 titers between times T1 and T2. When we calculated the ratio between T1 and T2, we found a significant decrease of all Abs (data not shown). When we repeated the analysis after separating HWs by their IgM seroprevalence, the tendency was similar except for IgG against S2 antigen among IgM − HWs (Fig. 5).

**Figure 6:**
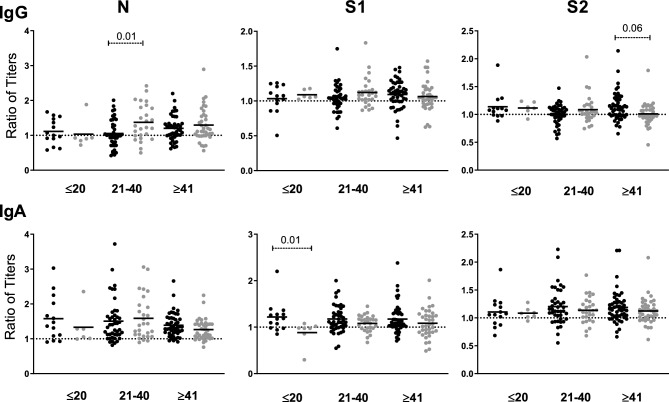
Distribution of ratios of titers T1/T2 according to time of infection (days). Dots represent the ratio of T1/T2 mean values (cut off value = 1). Values from IgM + HWs (black dots) and IgM—HWs (gray dots) were grouped by time (days) since infection. IgM + vs IgM—HWs titers were compared by Mann Whitney test (significant values are shown over discontinuous lines). Significant *p* value < 0.05.

It is interesting to note that, despite a generalized decrease of Ab titers, there was a significant proportion of samples that showed increased values on T2. The analysis of titer kinetics according to time since infection showed that IgM + HWs had a higher decrement of IgG anti-S2 when the T1 sample was collected ≥ 41d and IgA anti-S1 when T1 was < 21d. In addition, IgM − HWs showed a higher decrement of IgG anti-N in T1 samples collected 21–40 days after infection, as well as an increasing ratio for titers of the IgA anti S1 antigen (Fig. 6).

We could not find any association between kinetics and HWs’ characteristics or antecedents (data not shown). However, we found that IgM + HWs that reported dyspnea had higher titers of IgG anti-S2 in T2 than HWs without this symptom. In addition, IgM − HWs had a significant decrease of IgA anti-S1 in the group with cough and anti-S2 in the group without dyspnea (Supplementary Table 4). We repeated the analysis only with HWs with time (T1) since infection longer than 40 days and separated by IgM seroprevalence. IgM + HWs with cough showed a decreasing ratio of IgA against S1.

## Discussion

This study examined antibody titers in Hospital Workers (HWs) with mild COVID-19. IgM + samples had higher titers of IgA and IgG antibodies against specific antigens. Significant differences were found based on time since infection, sex, age, and symptoms. Antibody levels decreased over time but some samples showed increased values. Overall, the study provides insights into antibody dynamics in relation to COVID-19.

During the acute phase of COVID-19 infection, IgM antibodies are produced rapidly and they serve as the first line of defense against the virus. Typically, these antibodies can be detected within a week or two after the onset of symptoms. As patients transition into the convalescent phase, IgG antibodies are generated, offering more sustained protection against reinfection. IgG antibodies usually become detectable shortly after IgM antibodies, indicating a progression towards longer-term immunity. Since theoretically longer infection time implies IgM negativization, we categorized our samples based on the presence or absence of IgM in HWs’ blood samples. We unexpectedly found a significant proportion of HWs who remained IgM postive even beyond 40 days after reported infection suggesting considerable variability in the time it takes for IgM antibodies to clear from the system among individuals. Moreover, our analysis revealed notable serological disparities between IgM + and IgM − samples. IgM + samples had higher titers of IgG against N and IgA against S1 and S2 antigens than IgM − samples. On the other hand, IgM + samples collected < 21d had the lowest titers of IgG against N antigen but higher IgA against N titers, possibly indicating recent infection where IgG levels had not yet peaked. Our results are in concordance with previous reports of COVID-19 in which IgM levels were shown to appear first, followed by IgA and IgG, while Ab levels were detectable at approximately two weeks after onset of symptoms. It has been also shown that seroconversion for all Ig isotypes requires at least 6–10 days after onset of symptoms^15,18,19^. In others reports, there was a first IgA seroconversion at two days after onset of the initial symptoms and then IgM and IgG seroconversion at five days, with a median conversion time for IgA, IgM and IgG of 13 and 14 days, respectively^20–22^. Differences in COVID-19 severity and the demographic characteristics of cohorts could explain the complex kinetics of Abs found in different studies. Unlike many studies where serological analysis of IgG and IgM is conducted simultaneously, the focus on IgM positivity allowed us to discern unique patterns in antibody kinetics. All these findings suggest that in addition to the infection time, the exact timing of seroconversion can vary among individuals, influenced by factors such as disease severity and individual immune responses.

To our knowledge, there are few data that analyze correlations between SARS-CoV-2 Ab titers. Only one recent report has found significant correlations among Ab titers against S, N, M (membrane) antigens. Stronger correlations have previously been reported in severe forms^23^. Here, we found few significant correlations. Although mild COVID-19 patients produced lower titer of Abs, we found significant correlations, the most relevant of which were between titers of Abs against S1 and S2 in mild COVID-19. Our findings suggest that the production of certain Abs is more closely associated than others.

All our findings are consistent with previous studies on seroprevalence, in which there was high variability of Abs based on age, sex, type of institution, participant specific tasks and the incidence in the general population of each geographical area^24^. We found higher titers among male IgM − HWs of IgA against N and IgA anti S2. We also detected a tendency towards increasing Ab titers with age, especially IgA among IgM + HWs. In line with this, a lower seroprevalence has been reported in females with lower IgA responses against N and S2 antigens in a small cohort of patients^25,26^. Other reports showed lower Abs titers in young people than in older donors, and higher IgA levels against N total antigens in a cohort of patients older than 60 years^25,27,28^. It is possible that men and older people had prior contact with other coronaviruses and therefore developed faster, stronger or even cross-reacting immunity, reflected as higher Abs titers. However, many other factors could explain sex and age differences, such as hormone influence, immunity status and the presence of soluble blood proteins, as reported previously^29,30^. In this particular cohort, we were unable to identify differences in titers related to either antecedents or habits such as smoking among HWs. However, a systematic review of some studies has reported that smokers have a weakened immune response to SARS-CoV-2, including reduced levels of Abs^31^. The low frequency of smokers in our cohort of HWs may explain the different findings.

In agreement with our findings, some authors have suggested that the serological status of SARS-CoV-2 infection depends on vaccination against seasonal influenza because of vaccine-associated virus interferences. However, we found lower titers of IgG against S2 in HWs who had been previously vaccinated against influenza, and Stefanizzi P et al. showed that the titers of IgG anti-SARS-CoV-2 in HWs were more reduced in those receiving influenza + COVID-19 vaccines than in those receiving only COVID-19 vaccine^32^. Differences in methods, cohorts and antigens could explain the conflicting results.

In our cohort, HWs reported a miscellaneous spectrum of unspecific symptoms. The most frequent were myalgia and fever, followed by cough, anosmia and cephalea. All these symptoms were reported in outpatients as a general febrile syndrome and, according to the *Cochrane COVID‐19 Diagnostic Test Accuracy Group*^33^, any symptom can be considered pathognomonic as COVID-19 disease. Some of these symptoms were related to titers of immunoglobulins. Thus, high levels of IgG and IgA against SARS-CoV-2 N antigen were observed among IgM + HWs who had fever and cough, while anosmia and dyspnea were associated predominantly with lower titers of IgG and IgA anti-N and also IgA anti-S2. In a systematic review, the early testing for SARS-CoV-2 infection was usually carried out when anosmia, ageusia, fever or cough were reported^34^. However, most of these previous data correspond to severe hospitalized patients^35,36^. Our observations suggest that mild symptoms are related to responses with stronger and earlier Abs. Fever and levels of IgG antibodies in a virus infection can be attributed to factors such as the activation of the immune response and the release of pro-inflammatory molecules. On the other hand, the correlation between lower levels of antibodies and dyspnea in a virus infection can be attributed to factors such as the severity of the infection, impaired lung function, an exaggerated immune response, inflammation in the lungs, and the interplay of various immune factors. Previous reports have consistently shown that asymptomatic people were more likely to have greater IgA than IgG responses compared to those experiencing severe disease. Therefore, it seems probable that severe complications are related to lower serological Ab titers^37–39^. Lower antibody levels may indicate a weaker immune response, which can potentially lead to more severe respiratory symptoms and dyspnea. However, some authors have described that a robust IgA response may play a pathological role in SARS-CoV-2 infection and IgA at low levels may be able to control the infection. Based on all these findings, we can speculate that IgA contributes to early virus neutralization, leading to non-severe infection, while IgG contributes to longer term protection against more severe forms of disease. It is interesting to note that other studies have found that IgA and IgG are produced relatively late in the course of infection in severe disease^36,40–42^. The correlation between antibodies against a virus and symptoms during infection can be due to factors such as the immune response mounted against the virus, the level of viral replication and disease severity, individual variations in immune responses and host factors, the pathogenesis of the virus, and the formation of immune complexes. One aspect that we were unable to explore in our study was the relationship between the antibodies against SARS-CoV-2 and the duration of symptoms. Although we included a question about the duration of symptoms in the questionnaire, we found that the responses provided by participants were highly subjective and not consistently reliable. As a result, we were unable to incorporate this information into our analysis.

In our cohort of HWs, Ab levels declined over time and the dynamics of Ab titers were similar between IgM + and IgM − HWs. When the infection was recent (< 21d), there was a tendency to higher IgA and IgG titers. Reports consistent with our results showed that anti-SARS-CoV-2 Abs tended to decrease over the 6–13 months after infection. However, in some patients, titers were detectable beyond one year^7,43–45^. It is possible that the duration and intensity of the natural Ab response to SARS-CoV-2 varies according to disease severity and results in mild and severe patients are not comparable. Our results did not show different changes in Ab titers associated with HW antecedents or habits. On the other hand, patients with cough and anosmia had decreasing anti-N IgG and IgA levels. It is interesting to note that there was a proportion of patients with increasing titers at T2, especially when they reported dyspnea. The increasing titers in HWs with dyspnea may be a potential sign of worsening, as suggested by previous reports^39,45^. It would have been interesting to follow up the patients who went on to report dyspnea in order to observe their long-term outcomes.

Despite the detailed characterization of Ab titers and their short kinetics in mild COVID-19 patients, our study has several limitations. Participants were not randomly selected as a representation of diverse demographic characteristics, since they were all healthcare workers in a particular location. This fact may have introduced a bias in the study. We collected samples from positive HWs at only two post-infection time points, analyzing mildly symptomatic HW donors. Another drawback is that we did not analyze whether the new variants of the virus, with mostly asymptomatic patients and with a similar behavior or mild symptoms, demonstrate vaccine effectiveness^46,47^.

## Material and methods

### Study population

Between March and July, 2020, during the first main Covid-19 wave, 200 HWs from Hospital de la Santa Creu I Sant Pau were identified as COVID-19 positive, some of whom (N = 83) were tested by VIASURE SARS-CoV-2 Real Time PCR Detection Kit (CerTest Biotec SL, Zaragoza, Spain) or Cobas® SARS-CoV-2 Test (Roche, Basel, Switzerland), while the others (N = 117) were diagnosed when IgM anti-SARS-CoV-2 antibodies (for Nucleocapsid) were detected in baseline serum with NovaLisa® SARS-CoV-2 IgM (Gold Standard Diagnostics Group, Dietzenbach, Germany) and COVID-19 Coronavirus Rapid IgM and IgG Antibody Test Cassette (for Spike, SureScreen Diagnostics Ltd, Derby, UK), after the appearance of symptoms. Five negative IgM samples were excluded because infection was not confirmed by PCR or IgG serology. An electronic questionnaire developed specifically for this study, was performed to collect information on clinical and demographic variables. Data from clinical histories were also collected to obtain information about the symptomatology of the disease, antecedents, and the estimated duration of the infection. Identified positive cases were invited to participate in the study and to provide information about the presence of fever, acute respiratory symptoms (cough, sore throat, shortness of breath or dyspnea), loss or changed sense of smell (anosmia), headache, arthralgia, myalgia, fatigue, diarrhea, and skin rash. After signing consent, HWs were asked to donate serum twice after infection. In those with a molecular diagnosis, time of infection was considered at the moment of positive test. In those with serological diagnosis, the approximate time of infection was based on the initiation of symptoms. Despite some patients receiving molecular diagnosis, the time interval between the initial symptoms (and molecular diagnosis) and the T1 sample was found to be comparable to the time interval between symptom initiation and the T1 serum sample in individuals who underwent serological testing. Samples were collected at two time points since initial symptoms: T1 = 42 days (IQR, 34–51) and T2 = 103 days (IQR, 90–113) after infection. The time difference between samples was chosen arbitrarily (according to the disposition of the donor). Serum was then collected, processed and stored at -80ºC.

### Detection of IgG and IgA anti- SARS-CoV-2

Two ELISAs were performed to detect IgG and IgA against SARS-CoV-2 N, S1 and S2 antigens in all T1 and T2 samples. One was a commercial ELISA to detect IgG Nucleocapsid (N) (MBS398004 SARS-CoV-2 from MyBioSource Inc (San Diego, USA), which was performed according to the manufacturer’s instructions. The performance characteristics of the tests were: sensitivity 93.33%, specificity 93.89%. The second was an in-house ELISA, which was performed as per previous authors^13^. In-house ELISA was subjected to optimization with regards to dilution range, the use of blocking buffers, and variations in incubation times. The final conditions were chosen based on signal-to-noise (S/N) ratios. Briefly, previously validated recombinant SARS-CoV-2 proteins S1 (0.9 mg/dl) and S2 subunits (0.62 mg/ml) and Nucleocapsid Protein (1.05 mg/ml) (Raybiotech, Inc., Peachtree Corners, USA)^13^ were attached to wells on an ELISA plate. After overnight incubation at 4ºC, the wells were washed with PBS + Tween 0.05% and blocked for 1 h with PBS + 2% BSA. Serum samples (1:50 dilution for IgG and 1:20 dilution for IgA) were then added to wells and after 2 h of incubation, a secondary peroxidase conjugated affinity purified immunoglobulin was added: (a) 1:20,000 diluted goat anti-human IgG F(ab’)2 or (b) 1:2500 diluted goat anti-human IgA (alpha chain) (Rockland, Limerick, USA). Finally, a TMB substrate (BioLegend, San Diego, USA) generated a colored reaction, which was stopped with a stop solution of 2 M sulfuric acid. Absorbance (450–620 nm) was measured by spectrophotometer and the amount of SARS-CoV-2 IgG and IgA in the sample. Optical density was shown as O.D.

To validate the in-house ELISAs, that we have already applied to previous studies^13^, pre-pandemic samples served as negative controls. Cutoff was set at 95% percentile for pre-pandemic samples. Based on cutoff, the specificity of IgG anti-S1 and IgA anti-S1 were 88% and 85% respectively. The sensitivity of the assay was tested by analyzing samples from COVID-19 patients in concordance with SARS-CoV-2 IgG II Quant test (Abbott, Illinois, USA) and it was 85%. The intra-assay variation of IgG anti-S1 and IgA anti-S1 was evaluated by calculating the coefficient of variation (CV) of three different positive samples ten times within the same assay (10% and 10.2% respectively). In addition, the IgG anti-S1 levels of a sample from our cohort, obtained through the in-house ELISA, were compared with a commercial kit (NovaLisa® SARS-CoV-2, IgG anti-S1. NovaTec Immundiagnostica GmbH). The results obtained from both analyses exhibited a significant correlation (Supplementary Fig. 1, R = 0.475, *p* = 0.01).

### Statistical analysis

The Kolmogorov–Smirnov test was used to analyze the normal distribution of data. To describe our population, variables with a normal distribution were reported as mean ± standard error of the mean (s.e.m.) and the variables with a non-normal distribution were reported as median (interquartile range) (IQR). Comparisons between groups were tested with the student’s t or the Mann–Whitney test, according to Gaussian distribution. ANOVA and Kruskal–Wallis tests with Bonferroni correction were used for comparisons between more than two groups. Frequency comparisons were tested by chi-square. Correlation analyses were carried out with Pearson’s or Spearman correlations. Correlation matrixes were obtained employing an R package called “corrplot” (https://github.com/taiyun/corrplot). We used the GraphPad Prism 7.0 package and SPSS version 22, SPSS, Inc Chicago, Illinois, USA for this analysis. *p*-values < 0.05 were considered statistically significant. Binary logistic regression models were built to evaluate association between each symptom and anti-SARS-CoV2 antibodies (separated by specificity and titers). For these models, the variable selection was performed using stepwise regression. All statistical analyses were performed in SPSS.

### Ethics approval statement

The study was conducted according to the guidelines of the Declaration of Helsinki and approved by the Institutional Review Board (or Ethics Committee) of Research Institute of the Hospital de la Santa Creu i Sant Pau (HSCSP-IR) (protocol code of approval IIBSP-COV-2020-34).

### Supplementary Information

Supplementary Information.

## Data Availability

The datasets used and analysed during the current study available from the corresponding author on reasonable request.
