# Author Correction: Titers of IgG and IgA against SARS-CoV-2 proteins and their association with symptoms in mild COVID-19 infection

**DOI:** 10.1038/s41598-024-66711-9

**Published:** 2024-07-10

**Authors:** Andrés G. Abril, Jose Alejandre, Anais Mariscal, Leticia Alserawan, Nuria Rabella, Eva Roman, Joaquin Lopez‑Contreras, Ferran Navarro, Elena Serrano, Josep F. Nomdedeu, Silvia Vidal

**Affiliations:** 1 Departament Biologia Cel·lularFacultat de MedicinaFisiologia i Immunologia, Universitat Autònoma de Barcelona, Cerdanyola del Vallès, 08193 Bellaterra, Spain; 2 Althaia Xarxa Assistencial Universitària de Manresa, 08243 Manresa, Spain; 3 Institut de Recerca I Innovació en Ciències de la Vida i de la Salut a la Catalunya Central (IRIS-CC), 08500 Vic, Spain; 4 Grup de Malalties Inflamatòries, IIB-Sant Pau, Institut Recerca Hospital de la Santa Creu i Sant Pau, 08041 Barcelona, Spain; 5 Servei d’Immunologia, Hospital de la Santa Creu i Sant Pau, 08041 Barcelona, Spain; 6 Servei de Microbiologia, Hospital de la Santa Creu i Sant Pau, 08041 Barcelona, Spain; 7 Servei de Patologia Digestiva, Hospital de la Santa Creu i Sant Pau, 08041 Barcelona, Spain; 8 Servei de Malalties Infeccioses, Hospital de la Santa Creu i Sant Pau, 08041 Barcelona, Spain; 9 Biobanc IIB-Sant Pau, 08041 Barcelona, Spain; 10 Servei d’Hematologia, Hospital de la Santa Creu i Sant Pau, 08041 Barcelona, Spain

Correction to: *Scientific Reports* 10.1038/s41598-024-59634-y, published online 03 June 2024

The original version of this Article contained an error in the spelling of the author Josep F. Nomdedeu which was incorrectly given as Josep M. Nomdedeu.

The original Article has been corrected.

