# Supplementary material for: Titers of IgG and IgA against SARS-CoV-2 proteins and their association with symptoms in mild COVID-19 infection
Source: Sci Rep. 2024 Jun 3;14:12725. doi: 10.1038/s41598-024-59634-y (PMC11148197; doi:10.1038/s41598-024-59634-y)
Supplement: Supplementary file 1 — Supplementary Information. [file 41598_2024_59634_MOESM1_ESM.docx]

**Supplementary Table 1. Comparison of IgG and IgA titers**

**Supplementary Table 2. Correlation matrix of Ab titers in HWs with and without IgM anti-SARS-CoV2 antibodies**

| **IgM-** |  | | **IgG** | | **IgA** | | |
| --- | --- | --- | --- | --- | --- | --- | --- |
|  | **Antigens** | | **S1** | **S2** | **N** | **S1** | **S2** |
| **IgG** |  | **N** | **0.238**  ***** | 0.201  ***** | **0.495**  ******* | 0.157 | **0.318**  ****** |
|  |  | **S1** |  | **0.582**  ******* | 0.186 | **0.277**  ***** | **0.348**  ****** |
|  |  | **S2** |  |  | 0.118 | **0.231**  ***** | **0.408**  ******* |
| **IgA** |  | **N** |  |  |  | **0.244**  ***** | **0.264**  ***** |
|  |  | **S1** |  |  |  |  | **0.468**  ******* |

Pearson's correlation coefficients are shown, and significant values are highlighted in bold. (***p < 0.001, * p < 0.05).

**Supplementary Table 3. IgG and IgA titers according to infection symptoms.**

|  | **IgM+** | | | | | | **IgM -** | | | | | |
| --- | --- | --- | --- | --- | --- | --- | --- | --- | --- | --- | --- | --- |
|  | **IgG** | | | **IgA** | | | **IgG** | | | **IgA** | | |
|  | **N** | **S1** | **S2** | **N** | **S1** | **S2** | **N** | **S1** | **S2** | **N** | **S1** | **S2** |
| **FEVER** | **1.035**  **±0.095**  ****** | 0.672  ±0.024 | **0.503**  **±0.020**  ****** | **1.241**  **±0.079**  ****** | 0.588  ±0.041 | 0.787  ±0.041 | **0.815**  **±0.090**  ***** | 0.643  ±0.020 | 0.546  ±0.023 | **1.008**  **±0.082**  ***** | 0.552  ±0.037 | 0.666  ±0.033 |
|  | **0.681**  **±0.100** | 0.632  ±0.036 | **0.436**  **±0.015** | **0.878**  **±0.088** | 0.634  ±0.058 | 0.739  ±0.047 | **0.625**  **±0.094** | 0.658  ±0.032 | 0.510  ±0.031 | **0.816**  **±0.072** | 0.556  ±0.039 | 0.695  ±0.045 |
| **COUGH** | 0.893  ±0.093 | 0.673  ±0.032 | 0.501  ±0.021 | **1.177**  **±0.082**  ****** | 0.609  ±0.050 | 0.800  ±0.045 | **0.846**  **±0.080**  ****** | 0.626  ±0.027 | 0.540  ±0.024 | **1.085**  **±0.076**  ****** | 0.569  ±0.043 | **0.694**  **±0.035**  ***** |
|  | 0.754  ±0.089 | 0.609  ±0.027 | 0.457  ±0.018 | **0.945**  **±0.076** | 0.578  ±0.038 | 0.721  ±0.040 | **0.412**  **±0.065** | 0.660  ±0.027 | 0.529  ±0.028 | **0.674**  **±0.044** | 0.540  ±0.034 | **0.612**  **±0.027** |
| **ANOSMIA** | **0.721**  **±0.082** | **0.615**  **±0.027** | 0.480  ±0.021 | **1.017**  **±0.066** | 0.634  ±0.045 | 0.834  ±0.044 | 0.745  ±0.104 | 0.665  ±0.028 | **0.587**  **±0.026**  ****** | 0.901  ±0.064 | 0.539  ±0.044 | 0.692  ±0.038 |
|  | **1.055**  **±0.118**  ****** | **0.694**  **±0.036**  ***** | 0.475  ±0.018 | **1.158**  **±0.102**  ***** | 0.547  ±0.043 | 0.733  ±0.042 | 0.751  ±0.093 | 0.620  ±0.027 | **0.486**  **±0.025** | 0.861  ±0.073 | 0.560  ±0.034 | 0.627  ±0.027 |
| **PNEUMONIA** | 0.94  ±0.171 | 0.712  ±0.082 | 0.504  ±0.051 | **1.469**  **±0.132**  ****** | 0.595  ±0.090 | 0.877  ±0.097 | 1.042  ±0.254 | 0.574  ±0.044 | **0.416**  **±0.044** | 0.892  ±0.109 | 0.478  ±0.092 | 0.640  ±0.059 |
|  | 0.888  ±0.084 | 0.651  ±0.024 | 0.488  ±0.017 | **1.017**  **±0.061** | 0.581  ±0.032 | 0.762  ±0.031 | 0.707  ±0.069 | 0.657  ±0.022 | **0.547**  **±0.020**  ****** | 0.897  ±0.057 | 0.562  ±0.028 | 0.666  ±0.025 |
| **DYSPNEA** | **0.609**  **±0.110** | **0.479**  **±0.043** | **0.384**  **±0.033** | **0.784**  **±0.096** | **0.481**  **±0.058** | **0.609**  **±0.051** | 0.682  ±0.133 | 0.608  ±0.034 | 0.505  ±0.030 | 0.867  ±0.093 | 0.528  ±0.065 | 0.691  ±0.048 |
|  | **0.921**  **±0.086**  ***** | **0.659**  **±0.023**  ****** | **0.486**  **±0.014**  ****** | **1.104**  **±0.064**  ****** | **0.591**  **±0.032**  ***** | **0.782**  **±0.032**  ****** | 0.748  ±0.079 | 0.644  ±0.023 | 0.545  ±0.022 | 0.883  ±0.058 | 0.559  ±0.029 | 0.652  ±0.027 |
| **MYALGIA** | **0.911**  **±0.080**  ****** | 0.648  ±0.028 | 0.481  ±0.017 | 1.093  ±0.067 | 0.591  ±0.034 | 0.565  ±0.060 | **0.838**  **±0.103**  ***** | 0.624  ±0.024 | 0.544  ±0.025 | 0.888  ±0.065 | 0.542  ±0.035 | 0.639  ±0.028 |
|  | **0.638**  **±0.102** | 0.640  ±0.033 | 0.450  ±0.017 | 1.03  ±0.113 | 0.565  ±0.060 | 0.796  ±0.047 | **0.548**  **±0.065** | 0.664  ±0.029 | 0.524  ±0.027 | 0.869  ±0.076 | 0.566  ±0.040 | 0.663  ±0.036 |
| **CEPHALEA** | **0.933**  **±0.098**  ***** | 0.692  ±0.035 | 0.476  ±0.020 | 1.144  ±0.080 | 0.581  ±0.044 | 0.803  ±0.041 | 0.849  ±0.124 | 0.669  ±0.034 | 0.534  ±0.033 | 0.899  ±0.082 | **0.464**  **±0.053** | 0.695  ±0.040 |
|  | **0.699**  **±0.091** | 0.637  ±0.026 | 0.479  ±0.018 | 0.983  ±0.081 | 0.604  ±0.044 | 0.760  ±0.045 | 0.685  ±0.080 | 0.632  ±0.025 | 0.527  ±0.023 | 0.866  ±0.062 | **0.592**  **± 0.02**  ****** | 0.627  ±0.025 |
| **DIARRHEA** | **1.097**  **±0.139**  ****** | 0.678  ±0.036 | 0.450  ±0.023 | 1.166  ±0.096 | 0.546  ±0.041 | 0.758  ±0.050 | 0.874  ±0.131 | 0.643  ±0.030 | 0.557  ±0.033 | 0.907  ±0.090 | 0.521  ±0.047 | **0.556**  **±0.033** |
|  | **0.777**  **±0.084** | 0.650  ±0.031 | 0.464  ±0.017 | 1.026  ±0.073 | 0.622  ±0.043 | 0.794  ±0.039 | 0.686  ±0.080 | 0.638  ±0.025 | 0.517  ±0.023 | 0.891  ±0.064 | 0.564  ±0.033 | **0.711**  **±0.028**  ****** |

Mean O.D. values +/- s.e.m. are expressed with symptom presence (gray row) or symptom absence (white row). Statistically relevant differences (Kolmogorov-Smirnov test) are highlighted, and the highest values are represented (* p<0.05 or ** p<0.005). Green color represents significantly higher with symptom presence, Red color represents lower with symptom presence.

**Supplementary Table 4. Kinetics of IgG and IgA titers according to symptoms**

|  | **IgM+** | | | | | | **IgM -** | | | | | | |
| --- | --- | --- | --- | --- | --- | --- | --- | --- | --- | --- | --- | --- | --- |
|  | **IgG** | | | **IgA** | | | **IgG** | | | | **IgA** | | |
|  | **N** | **S1** | **S2** | **N** | **S1** | **S2** | **N** | **S1** | **S2** | **N** | | **S1** | **S2** |
| **FEVER** | 1.150 | 1.060 | 1.054 | 1.360 | 1.162 | 1.190 | 1.234 | 1.061 | 1.035 | 1.351 | | 1.060 | 1.060 |
|  | 1.079 | 1.060 | 1.058 | 1.352 | 1.142 | 1.082 | 1.119 | 1.075 | 1.031 | 1.412 | | 1.047 | 1.167 |
| **COUGHT** | 1.150 | 1.086 | 1.084 | 1.316 | 1.196 | 1.140 | 1.182 | 1.083 | 1.009 | 1.444 | | **1.131**  ***** | 1.132 |
|  | 1.108 | 1.034 | 1.042 | 1.423 | 1.120 | 1.151 | 1.197 | 1.053 | 1.050 | 1.326 | | **1.005** | 1.073 |
| **ANOSMIA** | 1.089 | 1.049 | 1.066 | 1.354 | 1.174 | 1.197 | 1.244 | 1.063 | 1.023 | 1.310 | | 1.080 | 1.114 |
|  | 1.160 | 1.060 | 1.033 | 1.385 | 1.135 | 1.127 | 1.147 | 1.071 | 1.038 | 1.467 | | 1.049 | 1.091 |
| **PNEUMONIA** | 1.163 | 1.119 | 1.090 | 1.275 | 1.282 | 1.134 | 1.270 | 1.106 | 0.924 | 1.440 | | 1.107 | 1.051 |
|  | 1.124 | 1.049 | 1.052 | 1.388 | 1.135 | 1.148 | 1.174 | 1.062 | 1.044 | 1.383 | | 1.057 | 1.109 |
| **DYSPNEA** | 1.067 | 1.012 | **0.981** | 1.349 | 1.146 | 1.072 | 1.319 | 1.044 | 0.964 | 1.274 | | 1.056 | **1.026** |
|  | 1.140 | 1.066 | **1.069 *** | 1.370 | 1.156 | 1.158 | 1.152 | 1.075 | 1.054 | 1.437 | | 1.064 | **1.131 *** |
| **MYALGIA** | 1.125 | 1.073 | 1.065 | 1.350 | 1.140 | 1.144 | 1.203 | 1.051 | 1.009 | 1.342 | | 1.055 | 1.072 |
|  | 1.142 | 1.028 | 1.042 | 1.409 | 1.185 | 1.148 | 1.170 | 1.085 | 1.056 | 1.449 | | 1.069 | 1.131 |
| **CEPHALEA** | 1.039 | 1.053 | 1.022 | 1.320 | 1.120 | 1.107 | 1.267 | 1.076 | 1.027 | 1.296 | | 1.081 | 1.090 |
|  | 1.272 | 1.064 | 1.091 | 1.418 | 1.188 | 1.184 | 1.143 | 1.061 | 1.033 | 1.453 | | 1.053 | 1.109 |
| **DIARRHEA** | 1.139 | 1.073 | 1.037 | 1.284 | 1.102 | 1.100 | 1.228 | 1.109 | 1.064 | 1.393 | | 1.045 | 1.064 |
|  | 1.121 | 1.050 | 1.069 | 1.432 | 1.181 | 1.171 | 1.169 | 1.049 | 1.015 | 1.388 | | 1.069 | 1.116 |

T1/T2 ratio is expressed in the table (cut off =1) with symptom presence (gray row) or symptom absence (white row). Values with statistical significance are highlighted and the highest value is represented (Kolmogorov-Smirnov test * p<0.05).

**Supplementary Figure 1**

Correlation of in-house ELISA values with commercial kit (NovaLisa) (Pearson correlation coefficient is shown).
